# The exciting and evolving journey of leadless pacing

**DOI:** 10.1093/eurheartjsupp/suae115

**Published:** 2025-03-24

**Authors:** Mikhael F El-Chami, Clemens Steinwender

**Affiliations:** Division of Cardiology-Section of Electrophysiology, Emory University School of Medicine, 550 Peachtree Street NE, Atlanta, GA 30308, USA; Department of Cardiology, Kepler University Hospital, Medical Faculty, Johannes Kepler University, Linz, Austria

Ten years ago, a leadless pacemaker (LP) [Micra transcatheter pacing system (TPS)] was implanted as part of the Micra investigational device exemption (IDE) study.^1^ This paved the way for Food and Drug Administration (FDA) approval in April of 2016. As part of an FDA mandate for any new technology, the Micra post-approval registry (PAR) was launched, and its results mimicked the IDE outcomes confirming the safety and efficacy of this device.^2^ The 5-year follow-up of this registry that enrolled ≅1800 implants was recently published with several important findings^3^:

- No device-related infections requiring LP removal were encountered.
- Low rate of revisions/upgrades 4.9% at 5 years.
- 2% rate of cardiac resynchronization therapy upgrade despite an 80% median pacing burden.

The Micra PAR is an ongoing registry with a 9-year follow-up planned. This long-term follow-up registry is a rarity in cardiovascular device studies and is a welcome step to ensure the long-term safety of this new technology.

The Micra coverage with evidence development (CED) study, an ongoing condition of coverage study required by the Center of Medicare and Medicaid Services, is prospectively enrolling all Medicare patients receiving a Micra LP and comparing the outcomes to patients implanted with a transvenous pacemaker (TV-PPM).^4^ The results of the Micra CED highlighted the long-term benefits of LP as compared to TV-PPM, confirming a recurrent theme with all Micra studies, a reduction in the rate of reintervention and complications with leadless pacing.^5–7^

The AVEIR-VR replaced the original Nanostim, an LP that encountered premature battery depletion and was pulled-off the market before meeting FDA requirements for approval.^8^ The LEADLESS II/Phase 2 study proved the efficacy and safety of the AVEIR-VR and led to the FDA approval in 2022. Hence, increasing the available options for single-chamber LP.^9^

Using the 3D accelerometer signals in the Micra LP, a mechanical signal that corresponds to atrial contraction can be sensed and tracked, allowing the introduction of an LP that can sense and track atrial activity.^10^ This expanded the number of patients who can benefit from an LP.^11^ With this device, atrioventricular (AV) synchrony is limited at faster heart rates, yet it can provide an alternative option to patients where AV synchrony at fast heart rate is not essential. Furthermore, improvement in sensing algorithms has increased the ability of Micra AV to track rates as fast as 135 b.p.m.

More recently, a dual-chamber LP, the AVEIR DR, was FDA approved based on the safety and efficacy results of the AVEIR i2i study.^12^ This is another milestone in the field of LP expanding the indications of LP to include patients with sinus node dysfunction.

A communicating LP (EMPOWER, Boston Scientific) with a subcutaneous Implantable Cardioverter Defibrillator (ICD) was recently evaluated in a clinical trial.^13^ This LP was implanted successfully and safely with the ability to communicate with an S-ICD to deliver anti-tachycardia pacing.

The last 10 years have seen several milestones in the field of LP. From the availability of a single-chamber LP by different manufacturers to a single LP that can function in the ‘VDD’ mode to finally a dual-chamber LP. The data behind single-chamber LP are robust with three main studies/registries (IDE, PAR, and CED) for Micra enrolling ≅9000 patients and two IDE studies for AVEIR (LEALESSII/Phase 2 and as part of the AVEIR i2i study) enrolling 500 patients. In our opinion, these data prove that single-chamber LP should be offered as an alternative to traditional TV-PPM while discussing the pros and cons of this technology and engaging in a shared decision-making with patients.

With all these exciting data, one could wonder what does the future hold? Should we approach LP in a ‘retro’ fashion and offer pacing as a modular concept? That is a patient with Sick Sinus Syndrome might only require an atrial LP, and a patient with AV block and intact sinus node might only need a ‘VDD’ device. If the aim is to minimize hardware in the heart, then the optimal device could be a single device that is able to sense and pace both the atrium and the ventricle.^14^ What if this device could also engage the conduction system and provide physiologic pacing? That might be the Holy Grail of pacing.

## Data Availability

No new data were generated or analysed in support of this research.
